# LncRNA‐DANCR contributes to lung adenocarcinoma progression by sponging miR‐496 to modulate mTOR expression

**DOI:** 10.1111/jcmm.13420

**Published:** 2017-12-20

**Authors:** Qing‐chun Lu, Zhuang‐hua Rui, Zhong‐liang Guo, Wang Xie, Shan Shan, Tao Ren

**Affiliations:** ^1^ Department of Respiratory Medicine Shanghai East Hospital Tongji University School of Medicine Shanghai China; ^2^ Department of Respiratory Medicine Shanghai Jiao Tong University Affiliated Sixth People's Hospital China

**Keywords:** lung adenocarcinoma, DANCR, miR‐496, mTOR, ceRNA, sponge

## Abstract

Long non‐coding RNAs (lncRNAs) have emerged as new and important regulators of pathological processes including tumour development. In this study, we demonstrated that differentiation antagonizing non‐protein coding RNA (DANCR) was up‐regulated in lung adenocarcinoma (ADC) and that the knockdown of DANCR inhibited tumour cell proliferation, migration and invasion and restored cell apoptosis rescued; cotransfection with a miR‐496 inhibitor reversed these effects. Luciferase reporter assays showed that miR‐496 directly modulated DANCR; additionally, we used RNA‐binding protein immunoprecipitation (RIP) and RNA pull‐down assays to further confirm that the suppression of DANCR by miR‐496 was RISC‐dependent. Our study also indicated that mTOR was a target of miR‐496 and that DANCR could modulate the expression levels of mTOR by working as a competing endogenous RNA (ceRNA). Furthermore, the knockdown of DANCR reduced tumour volumes *in vivo* compared with those of the control group. In conclusion, this study showed that DANCR might be an oncogenic lncRNA that regulates mTOR expression through directly binding to miR‐496. DANCR may be regarded as a biomarker or therapeutic target for ADC.

## Introduction

Lung cancer is the most common cancer and remains the leading cause of cancer‐related death in the world 1. Lung ADC is the most common type of lung cancer, accounting for almost half of all lung cancer cases 2, 3. Although multimodal diagnostic methods and treatments have been implemented in the past years, ADC is still a lethal disease, with a 5‐year survival rate of approximately 15% 4. Hence, further investigation of the aetiology and biological mechanisms of ADC is important for developing novel therapeutic targets and biomarkers.

lncRNAs are a class of non‐protein‐coding RNAs longer than 200 nucleotides that are poorly conserved in different species 5. In the past years, studies have implicated that lncRNAs functioned as regulators of numerous pathological processes, such as cancer progression, metastasis and tumour drug resistance 6, 7, 8. Several lncRNAs have been reported to be involved in lung cancer tumorigenesis. For example, the lncRNA MALAT‐1 was reported to be extremely abundant in lung cancer and was identified as a prognostic marker 9. Another lncRNA, NEAT1, was identified to function as an oncogene by acting as a ceRNA in NSCLC 10. However, the precise functional roles of lncRNAs in lung cancer still need clarification.

Differentiation antagonizing non‐protein coding RNA (DANCR), also named ANCR, was reported to be widely distributed in human organs and was first identified as an RNA related to progenitor cell differentiation‐related RNA 11. In recent years, many studies have been conducted to evaluate the function of DANCR in biological processes, including stem cell differentiation, cell proliferation and cancer progression 12. The modulatory role of DANCR in the progression of several types of cancer, including colorectal cancer, hepatocellular carcinoma, prostate cancer and breast cancer, has already been studied 13, 14, 15, 16. However, the role of DANCR in lung cancer still needs to be explored and its function remains to be characterized.

In this study, we explored the potential involvement of DANCR in lung ADC. We found that the expression level of DANCR is up‐regulated in cancer tissues compared with that in adjacent normal tissues. When DANCR was knocked down, cancer cell proliferation, migration and invasion were inhibited, and cell apoptosis was increased. Additionally, our study suggested that DANCR may regulate mammalian target of rapamycin (mTOR) expression through sponging miR‐496, thus modulating tumour growth. In conclusion, these results indicate that DANCR regulates lung ADC progression by working as a ceRNA to regulate mTOR by sponging miR‐496.

## Materials and methods

### Patients and samples

From January 2013 to February 2017, a total of 34 lung ADC tissue samples and their matched adjacent normal lung tissue samples were collected at Shanghai East Hospital (Tongji University, School of Medicine). The adjacent normal lung tissue samples were >3 cm away from the edge of the tumour, and no tumour cells were observed in these samples. The samples were snap‐frozen in liquid nitrogen and stored until RNA extraction was performed. All samples were diagnosed by a pathologist and histologically confirmed as ADC. The patients were staged according to the TNM staging system. All the patients recruited were diagnosed as stage II, with a tumour size <3 cm and with lymph node metastasis. The study was approved by the Human Ethics Committee of Shanghai East Hospital at Tongji University (Shanghai, China).

### Cell culture

Three human ADC cell lines (A549, H1299 and H358), human embryonic kidney (HEK) 293T cells and a human bronchial epithelial cell line (HBE) were used in this study. All ADC cell lines and 293T cell line were purchased from the cell bank of the Chinese Academy of Science (Shanghai, China), and the HBE cell line was supplied by Prof. Bailing Luo from Xiangya Hospital (Changsha, China). The cells were maintained in DMEM (Hyclone, Camarillo, CA, USA) supplemented with 10% foetal bovine serum (Gibco, Grand Island, NY, USA), 100 μg/ml penicillin and 100 μg/ml streptomycin (Sigma, St. Louis, MO, USA). Then, the cells were incubated in at 37°C and 5% CO_2_.

### RNA isolation, reverse transcription and qRT‐PCR

Total RNA from the cell lines and lung tissues was extracted using Trizol reagent (Invitrogen, Camarillo, CA, USA) according to the manufacturer's instructions. After extraction, the RNA samples and miRNA samples were reverse‐transcribed into cDNA using a RevertAid RT Reverse Transcription Kit (Thermo, Waltham, MA, USA) or miRNA Reverse Transcription Kit (Tiangen, Beijing, China), respectively. The cDNA templates were amplified by qRT‐PCR using SYBR Green Mix (Thermo). GAPDH was used as an internal control for lncRNAs, and miRNA samples were normalized to U6 expression. The primer sequences used were as follows: GAPDH‐F, 5′‐GAGTCAACGGATTTGGTCGT‐3′; GAPDH‐R, 5′‐TTGATTTTGGAGGGATCTCG‐3′; DANCR‐F, 5′‐GCCACTATGTAGAGGGTTTC‐3′; DANCR‐R, 5′‐ACCTGCGCTAAGAACTGAGG‐3′; mTOR‐F, 5′‐ATGCTTGGAACCTGACCTG‐3′; mTOR‐R, 5′‐TCTTGACTCATCTCTCGGAGTT‐3′; miR‐496‐F, 5′‐CGCTGAGTATTACATGGCCAATCTC‐3′; the reverse primer of miR‐496 was supported by Tiangen company. The relative fold expression levels were analysed using the 2^−▵▵Ct^ method.

### RNAi and cell transfection

The DANCR‐siRNA, RNA mimics, inhibitors and negative control were all synthesized by GenePharma (Shanghai, China). The shRNAs inserted into the lentivirus and the DANCR overexpression plasmid were purchased from OBiO Biology (Shanghai, China). The siRNA sequences were as follows: si‐DANCR, forward: 5′‐GCUGGUAUUUCAAUUGACUTT‐3′,reverse: 5′‐AGUCAAUUGAAAUACCAGCTT‐3′; negative control: forward: 5′‐UUCUCCGAACGUGUCACGUTT‐3′, reverse: 5′‐ACGUGACACGUUCGGAGAATT‐3′; LV‐sh‐NC, forward: 5′‐ TTCTCCGAACGTGTCACGT‐3′, reverse: 5′‐AAGAGGCTTGCACAGTGCA‐3′; LV‐sh‐DANCR, forward: 5′‐GCTGGTATTTCAATTGACTTT‐3′, reverse: 5′‐AGTCAATTGAAATACCAGCTT‐3′. The RNAi experiments were performed using the transfection reagent Lipofectamine 3000 (Invitrogen). The working concentration of siRNA was 20 nM and that for RNA mimics and inhibitors was 50 nM. The concentration used for plasmids was 100 nM.

### RNA‐binding protein immunoprecipitation assay

The RIP assays were performed according to the manufacturer's instructions (Magna RIP™ RNA‐Binding Protein Immunoprecipitation Kit; Millipore, Billerica, MA, USA). Briefly, A549 cells were cultured to 90% confluence and harvested using RIP lysis buffer. At the same time, human anti‐Ago2 antibody (Abcam, Cambridge, MA, USA) was incubated with lysis buffer containing magnetic beads to conjugate the antibody to the magnetic beads. The beads were attracted by a magnetic separator, and proteinase K was added to digest the protein. Finally, the supernatants containing RNA were collected and extracted using Trizol reagent. The negative control was normal rabbit IgG provided in the RIP Kit.

### RNA pull‐down assay

RNA pull‐down assays were performed to determine whether DANCR was directly associated with miRNA. To confirm this, a biotin‐labelled miR‐496 mimic was designed and transfected into A549 cells as previously reported 17. Twenty‐four hours later, the cells were collected and incubated with M‐280 streptavidin magnetic beads (Invitrogen) at 4°C for 4 hrs with rotation. After the 4‐h incubation, the beads were washed with lysis buffer containing proteinase K (Invitrogen) and 10% SDS, and the supernatants were collected. RNA was isolated by combining the supernatants with acid‐phenol:chloroform. PCR assays were used to detect the coprecipitated RNA. The miR‐67 mimic of *C.elegans* was used as a negative control.

### Luciferase reporter assay

Cells were cultivated in 96‐well plates and transfected with 0.5 μg of empty pMIR‐REPOR NC, pMIR‐REPOR DANCR‐wt or pMIR‐REPOR DANCR‐mut (OBiO Biology, Shanghai), as well as miR‐496 mimic or NC mimic. Following transfection for 48 hrs, luciferase activity was measured using a dual‐luciferase reporter assay system (Promega, Madison, WI, USA). The Renilla luciferase activity was used as an internal control for normalization.

### Cell proliferation assay

The cell proliferation rate was detected using Cell Counting Kit‐8 (CCK‐8; Dojindo, JPN) and 5‐ethynyl‐2′‐deoxyuridine assays (EdU; Ribobio, Guangzhou, China). A549 and H1299 cells were transfected with si‐NC, si‐DANCR, miR‐496 inhibitor or si‐DANCR + miR‐496 inhibitor, harvested and seeded into 96‐well plates. After 24, 48 72 or 96 hrs, 10 μl of CCK‐8 assay reagent was added to each well, incubated for 2 hrs and then measured using an enzyme immunoassay analyser (Bio‐rad, Hercules, CA, USA). The EdU assay was performed according to the manufacturer's instructions. Briefly, cells were cultured in 96‐well plates and incubated with 50 μM EdU for 2 hrs. Then, the cells were washed with PBS three times, fixed in 4% paraformaldehyde and incubated with 2 mg/ml glycine, followed by Apollo reaction cocktail. After 30 min., Hoechst 33,342 was added to the fixed cells and incubated for half an hour. The cells were observed under a microscope, and the proliferation rate was calculated.

### Cell apoptosis assay

Cells were transfected and cultured in a six‐well plate before flow cytometry analyses were performed. After a 48‐h incubation, cells were digested, stained with AnnexinV‐PE from a cell apoptosis kit (BD Bioscience, San Jose, CA, USA) and analysed using a flow cytometer to determine the cell apoptosis rate.

### Wound‐healing experiment

A p200 pipet tip was used to create a scratch when the cells reached 100% confluence. Then, the cells were washed with PBS three times and incubated with DMEM without serum. At 0 and 24 hrs, images were taken using a microscope, and the data were collected and analysed to determine the migration of the cells.

### Cell migration and invasion assay

The capability of cell migration and invasion was investigated by transwell assay. Cells were seeded into the upper chambers of a transwell plate (Corning, Cambridge, MA, USA) with or without Matrigel (Corning) in 200 μl of serum‐free DMEM, and the lower chambers were filled with 600 μl of DMEM containing 10% FBS. After 24 hrs of incubation, the filters were treated with 4% paraformaldehyde for 10 min. and then stained with crystal violet for another 10 min. The number of cells was counted and calculated.

### Western blot analysis

Whole cell extracts were harvested using RIPA buffer (Beyotime, Shanghai, China). The supernatants were collected and measured using a BCA protein assay kit (Beyotime). The protein extracts were separated using 8% SDS‐PAGE gels, transferred to polyvinylidene fluoride (PVDF) membranes (Millipore) and probed with an antibody against mTOR (1:1000; CST, USA). At 24 hrs later, the membranes were washed three times, incubated with a secondary antibody (1:5000; CST, USA) and visualized by enhanced chemiluminescence (ECL) (Millipore, USA). GAPDH (1:2000; CST, USA) was used as an internal control.

### Xenograft mouse model

A549 cells stably transfected with lv‐shRNA‐NC or lv‐shRNA‐DANCR were injected subcutaneously into 8‐week‐old male nude mice (*n* = 5 mice per group). The sizes of the tumours were measured every week. After 4 weeks, the mice were killed, the tumour tissues were excised, and immunohistochemical analyses were performed to determine mTOR expression. The animal experiments were performed according to the Guide for the Care and Use of Laboratory Animals published by the US National Institutes of Health.

### Immunohistochemical analysis

The tumour samples were embedded in paraffin after fixation in 4% paraformaldehyde. Then, the specimens were incubated at 4°C overnight with the mTOR primary antibody (1:250, Abcam); subsequently, the samples were treated with secondary antibody and stained with diaminobenzidine. The results were evaluated by two pathologists individually.

### Statistical analysis

All the statistical data are presented as the means ± S.D. Two‐tailed Student's *t*‐test or one‐way anova was performed for comparisons between groups. Expression correlation assays were analysed using Pearson's coefficient correlation. A value of *P* < 0.05 was considered to be statistically significant.

## Results

### DANCR is up‐regulated in lung ADC

**Figure 1:**
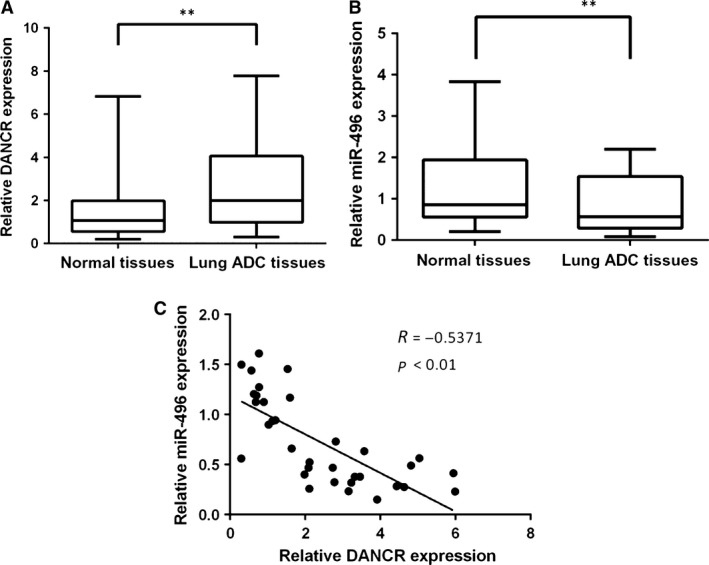
The expression levels of DANCR and miR‐496 in lung ADC tissues. (**A**) The expression level of DANCR in 34 pairs of samples of lung ADC tissues and their adjacent normal tissues was evaluated by qRT‐PCR. The expression of DANCR was normalized to that of GAPDH. (**B**) The expression level of miR‐496 in lung ADC tissues and adjacent counterparts, and the expression of miR‐496 were normalized to that of the housekeeping gene U6. (**C**) The correlation between DANCR and miR‐496 was evaluated (*R* = −0.5371). **P < 0.05.

**Figure 2:**
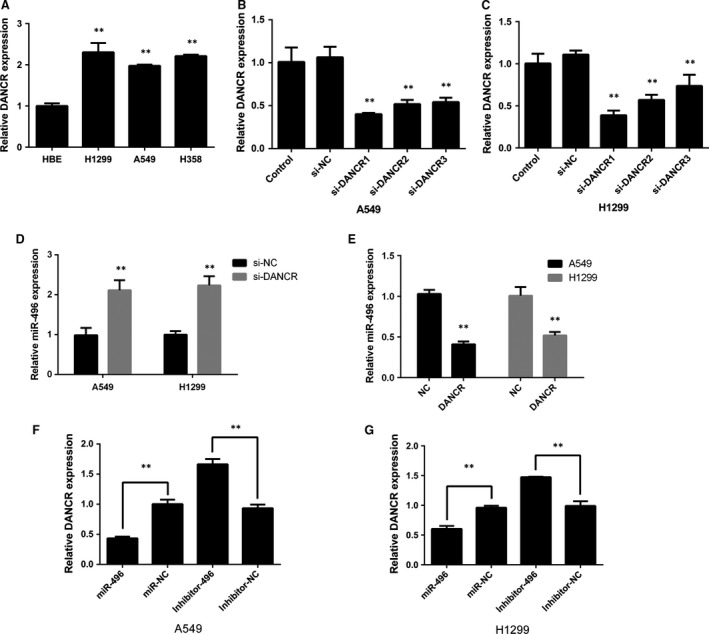
The reciprocal repression effect of lncRNA‐DANCR and miR‐496. (**A**) The expression level of DANCR was detected in three ADC cell lines and the HBE cell line. DANCR expression was compared among these cell lines. (**B** and **C**) A549 and H1299 cell lines were transfected with three DANCR siRNAs. (**D** and **E**) Expression levels of miR‐496 in A549 and H1299 cells after the overexpression or down‐regulation of DANCR. (**F** and **G**) qPCR analysis of DANCR after cells were transfected with miR‐496 mimic or inhibitor for 24 hrs. **P < 0.05.

We collected 34 pairs of human lung ADC tissues and their adjacent normal tissues to evaluate the expression levels of DANCR in lung cancer. QRT‐PCR assays revealed that DANCR expression was increased in ADC tissues compared with that in the adjacent normal tissues (Fig. 1A, *P* < 0.05). Then, the expression levels of DANCR were evaluated in three ADC cell lines (A549, H1299 and H358) and the HBE cell line; DANCR expression was significantly increased in all three cancer cell lines (Fig. 2A). We used A549 and H1299 cells in the following experiments; these cell lines were transfected with three different DANCR siRNAs. Of the three DANCR siRNAs, si‐DANCR‐1 was chosen because its efficiency was the best (Fig. 2B and C).

### Reciprocal modulation of DANCR and miR‐496

One of the intrinsic mechanisms of lncRNA is competition for endogenous RNA (ceRNA). In this study, we explored the underlying mechanism of DANCR in the development of ADC. A bioinformatics analysis was performed using Starbase v2.0 (http://starbase.sysu.edu.cn/) and a cohort of seven potential miRNAs targeting DANCR was predicted. The expression profiles of these miRNAs in DANCR‐knockdown A549 cells were examined by qRT‐PCR. The results revealed miR‐496 as a candidate miRNA because it was up‐regulated more than twofold in response to silence of DANCR (Table 1). To confirm this, the expression levels of DANCR and miR‐496 were examined in cancer tissues. The results showed that miR‐496 was down‐regulated in tissues that expressed a high level of DANCR (Fig. 1B). Interestingly, DANCR expression was negatively correlated with miR‐496 expression (Fig. 1C).

**Table 1:** Fold change expression of seven predicted miRNAs in A549 cells transfected with si‐DANCR

miRNAs	Fold change	*P* value
miR‐135a‐5p	1.33	<0.01
miR‐33b‐5p	1.21	<0.01
miR‐758‐3p	2.04	<0.01
miR‐216a‐5p	1.55	<0.01
miR‐33a‐5p	0.80	<0.01
miR‐496	2.61	<0.01
miR‐135b‐5p	1.56	<0.01

The relationship between DANCR and miR‐496 was further investigated by overexpressing or inhibiting DANCR expression in the cell lines. After knocking down DANCR, miR‐496 expression was increased (Fig. 2D). In contrast, the overexpression of DANCR significantly suppressed the levels of miR‐496 (Fig. 2E). Next, we determined whether miR‐496 affected DANCR. miR‐496 mimic or inhibitor was transfected into A549 and H1299 cells, and the DANCR levels were measured (Fig. 2F and G). The results showed that when the cells were transfected with miR‐496 mimic, DANCR expression was decreased, whereas high levels of DANCR were observed in the inhibitor‐transfected cells.

### DANCR is a direct target of miR‐496

**Figure 3:**
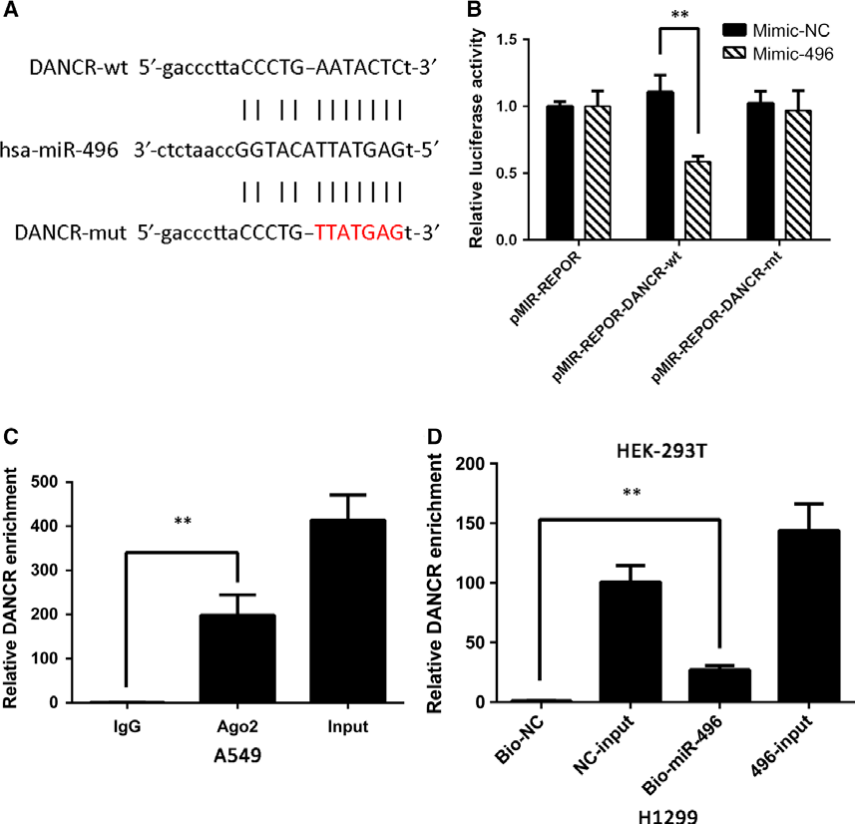
DANCR and miR‐496 directly target each other. (**A**) Binding site of miR‐496 and DANCR as predicted by Starbase v2.0. (**B**) Relative luciferase activity in HEK‐293T cells transfected with miR‐496 mimic or mimic‐NC and cotransfected with the control vector, pMIR‐REPOR‐DANCR‐wt or pMIR‐REPOR‐DANCR‐mut. (**C**) RIP assays were conducted to evaluate the amount of DANCR bound to Ago2. (**D**) Mount of DANCR bound to biotin‐labelled miR‐496 mimic or biotin‐labelled negative control. RNA pull‐down assays were performed after 24 hrs of transfection. **P < 0.05.

To further analyse whether DANCR is a direct target of miR‐496, a dual‐luciferase reporter assay was performed. First, a luciferase plasmid containing the entire DANCR sequence was synthesized; then, a mutant reporter harbouring a mutation in the predicted binding site for miR‐496 was designed as a negative control. Luciferase assay results indicated that miR‐496 mimic significantly decreased luciferase reporter expression in the cells transfected with pMIR‐REPOR DANCR‐wt but not those transfected with DANCR‐mut or NC (Fig. 3A and B).

It is commonly known that miRNA functions by regulating RNA‐induced silencing complex (RISC) 18. Ago2 is an important component of RISC that plays an essential role in RNA cleavage. Therefore, we conducted RIP assays to investigate whether miR‐496 modulates DANCR through RISC formation. The results showed that compared with the negative control (beads incubated with rabbit IgG), DANCR was preferentially enriched in anti‐Ago2 antibody‐incubated beads (Fig. 3C). RNA pull‐down assays were also performed to determine whether DANCR and miR‐496 bind to each other. As the results show, the biotin‐labelled miR‐496 mimic pulled down more DANCR lncRNA than the negative control (Fig. 3D). These experiments demonstrated that DANCR bound to miR‐496 directly.

### DANCR and miR‐496 effects on cell proliferation, migration and invasion

**Figure 4:**
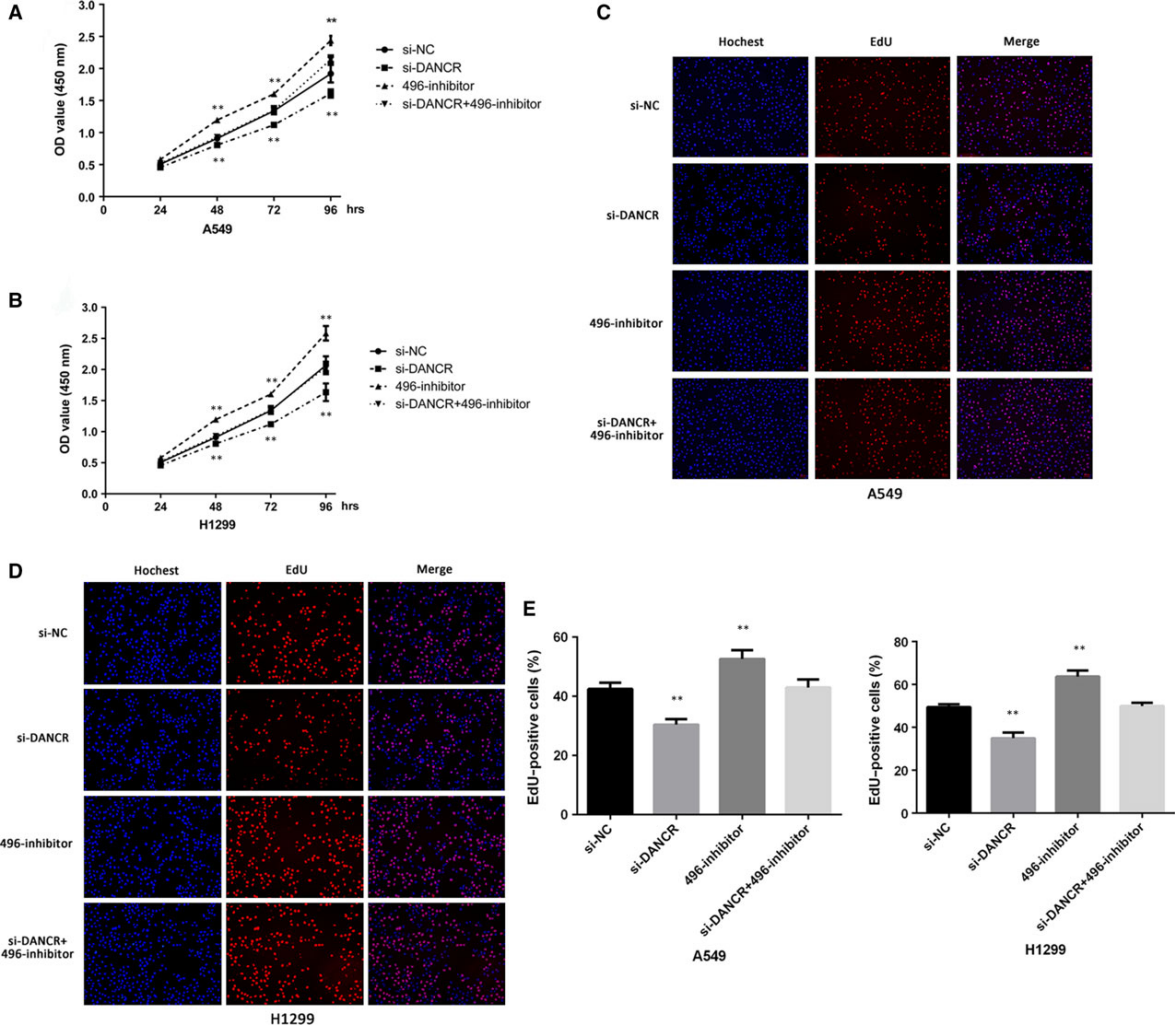
The role of DANCR and miR‐496 in cell proliferation. (**A** and **B**) CCK‐8 cell viability assays were used to evaluate the si‐NC, si‐DANCR, miR‐496 inhibitor and si‐DANCR+miR‐496 inhibitor groups. (**C**–**E**) EdU assays were used to determine cell proliferation. The cell proliferation rates in A549 and H1299 cells transfected with si‐NC, si‐DANCR, miR‐496 inhibitor or si‐DANCR+miR‐496 inhibitor were calculated. **P < 0.05.

**Figure 5:**
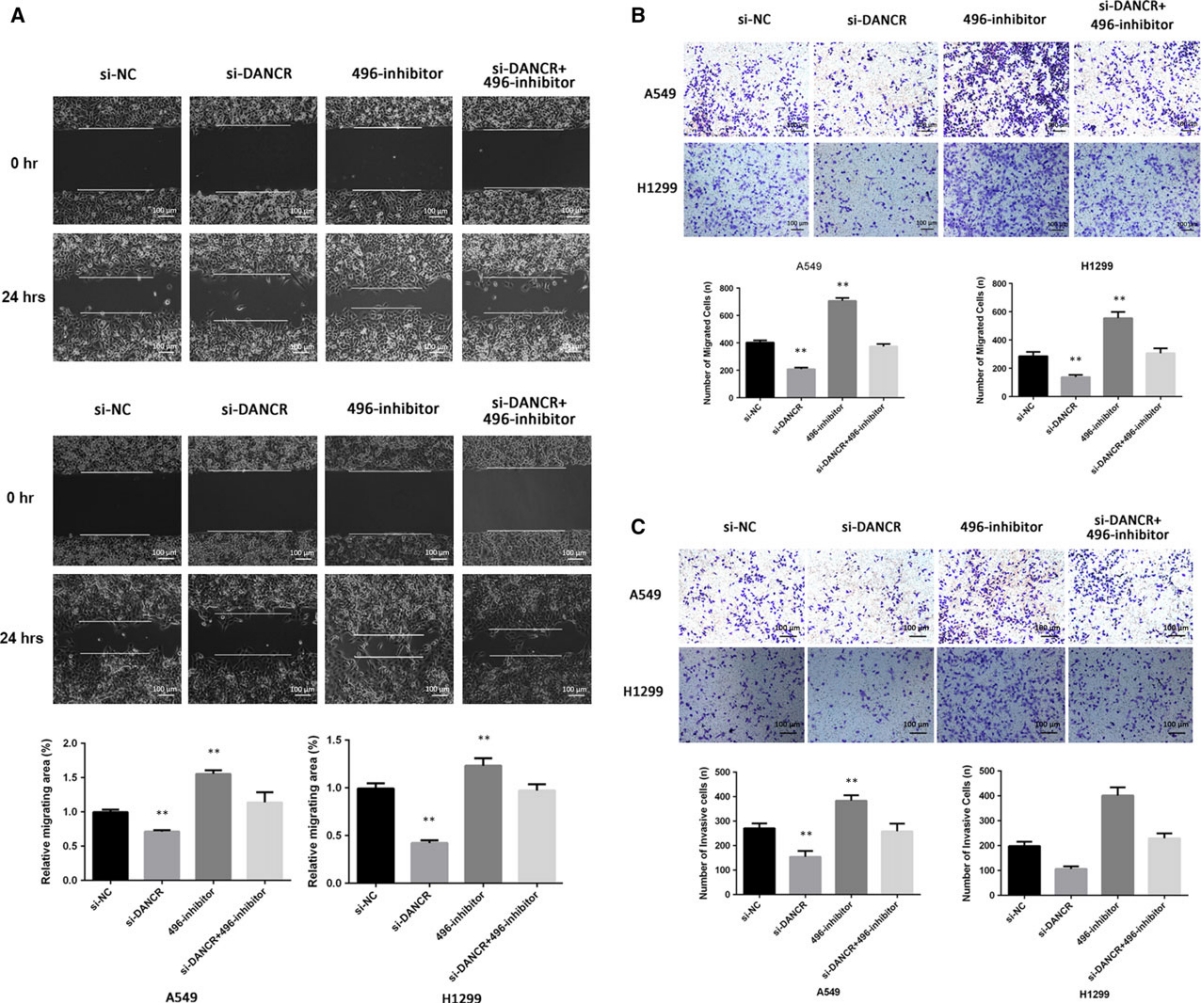
The role of DANCR and miR‐496 in cell migration and invasion. (**A**) Wound‐healing assays were conducted to detect the cell migration ability of each group. (**B**) A549 and H1299 cells were transfected with si‐NC, si‐DANCR, miR‐496 inhibitor or si‐DANCR+miR‐496 inhibitor. After 24 hrs, transwell migration assays were performed. (**C**) Transwell invasion assays were performed, and the number of invasive cells was calculated. **P < 0.05.

Next, we explored the effects of DANCR and miR‐496 on NSCLC cell proliferation, migration and invasion. The number of cells in the si‐DANCR group was significantly reduced compared with that in the NC group, but the inhibitory effect was reversed when the cells were cotransfected with si‐DANCR and the miR‐496 inhibitor. Transfection with the miR‐496 inhibitor alone slightly increased cell proliferation (Fig. 4A–E). We also examined whether DANCR affected lung cancer cell migration and invasion. Wound‐healing and transwell assays suggested that treatment with si‐DANCR could suppress both NSCLC cell migration and invasion abilities. Similar to above, these effects were also reversed when si‐DANCR and the miR‐496 inhibitor were cotransfected (Fig. 5A–C).

### DANCR inhibited cell apoptosis by competing with miR‐496

**Figure 6:**
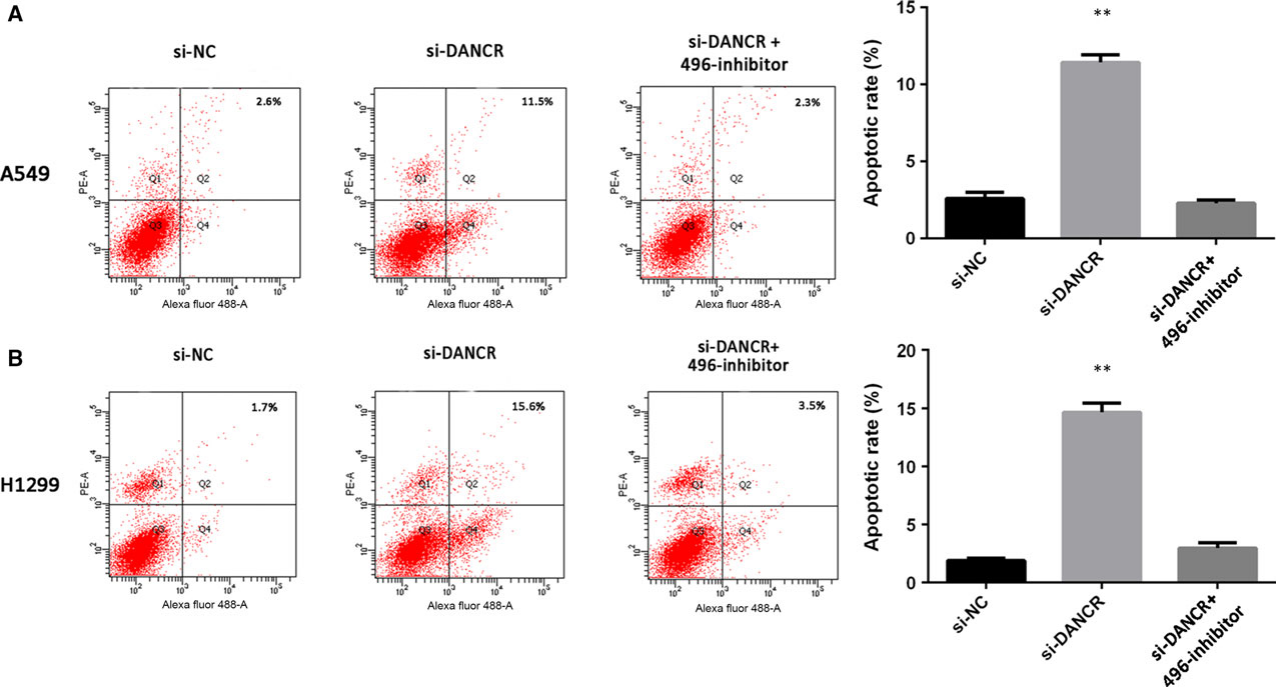
The role of DANCR and miR‐496 in cell apoptosis. (**A** and **B**) Flow cytometry analyses were used to determine apoptosis after cells were transfected with si‐NC, si‐DANCR, miR‐496 inhibitor or si‐DANCR+miR‐496 inhibitor. **P < 0.05.

We continued to investigate the effects of DANCR on cell apoptosis and used flow cytometry, to measure the proportion of cells undergoing apoptosis. The results indicated that the cell apoptosis rate was increased after treatment with si‐DANCR, whereas the miR‐496 inhibitor prevented cell apoptosis (Fig. 6A and B).

### DANCR inhibited the expression of miR‐496 and increased that of mTOR

**Figure 7:**
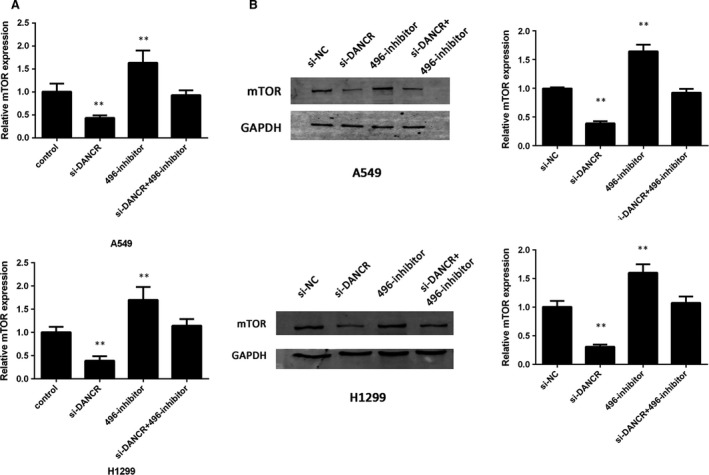
DANCR modulates the expression of mTOR. (**A**) mRNA levels of mTOR in A549 and H1299 cells transfected with si‐NC, si‐DANCR, miR‐496 inhibitor or si‐DANCR+miR‐496 inhibitor. (**B**) Protein levels of mTOR in A549 and H1299 cells transfected with si‐NC, si‐DANCR, miR‐496 inhibitor or si‐DANCR+miR‐496 inhibitor. **P < 0.05.

mTOR plays a crucial role in tumorigenesis, and it has been reported as a target of miR‐496 19. *In vitro* assays, both qRT‐PCR and Western blots showed that the knockdown of DANCR significantly reduced the expression of mTOR at the transcription and translation levels (Fig. 7A and B). These effects were reversed by the miR‐496 inhibitor, which indicated that DANCR could modulate mTOR, partially by acting as a ceRNA (Fig. 7A and B). These results showed that DANCR might increase mTOR expression by binding to miR‐496.

### Knockdown of DANCR prevented tumour growth and decreased mTOR *in vivo*

**Figure 8:**
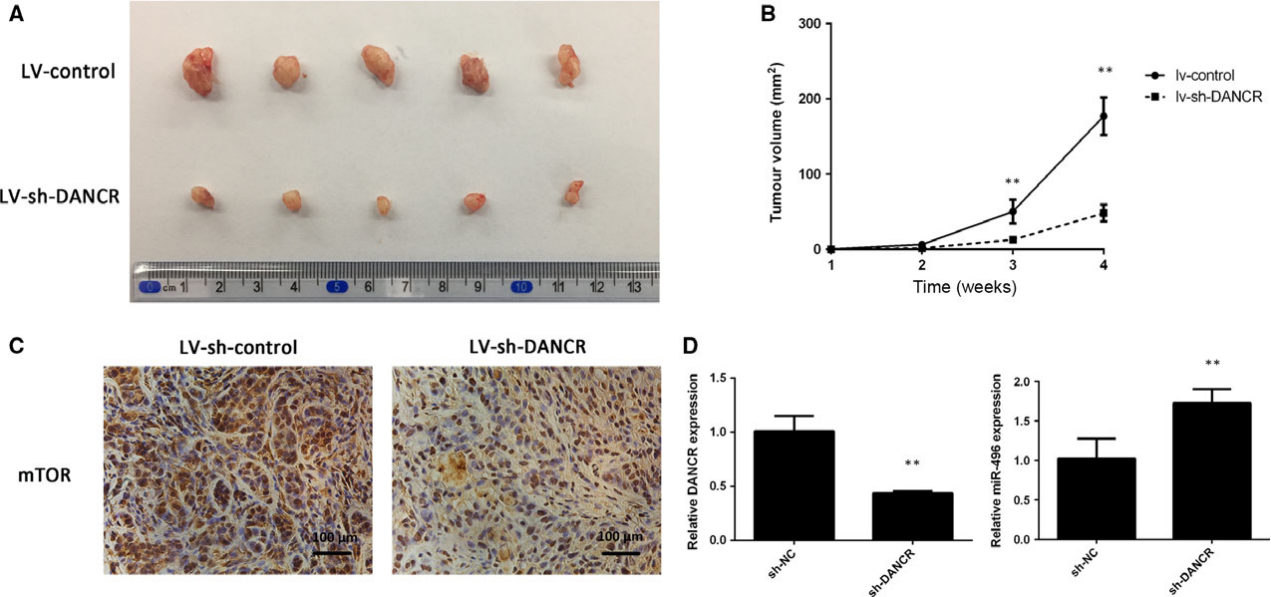
Knockdown of DANCR inhibits tumour growth *in vivo*. (**A**) The tumour volumes in the lv‐shRNA‐DANCR and lv‐shRNA‐NC groups were measured at 4 weeks after injection. (**B**) The tumour volumes of the mice were measured every week after the cell injection. (**C**) At 4 weeks later, the mice were killed, and the tumour tissues were excised. The mTOR expression in the tumour tissues was detected by IHC method in the lv‐shRNA‐DANCR and lv‐shRNA‐NC group. (**D**) Expression levels of DANCR and miR‐496 in tumours. **P < 0.05.

To verify the *in vitro* findings, a lv‐sh‐DANCR lentivirus was designed and transfected in A549 cells to construct a stable cell line in which DANCR was silenced. Then, the cells were injected subcutaneously into nude mice. The tumour volumes of the lv‐sh‐DANCR group were much smaller than those of the lv‐control group (Fig. 8A and B). IHC was performed to determine the expression of mTOR in the tumours. The expression of mTOR was suppressed by the knockdown of DANCR, which was in accordance with the *in vitro* results (Fig. 8C). Furthermore, PCR assays of the resected tumour tissues showed that DANCR expression was negatively associated with miR‐496 (Fig. 8D). These results demonstrate that DANCR positively regulates NSCLC cell proliferation *in vivo* and that it may act as a ceRNA to modulate mTOR.

## Discussion

It is widely believed that lncRNAs regulate complex biological processes, including tumorigenesis 20. A new lncRNA, DANCR, has been reported to function as a regulator of different tumour types, but its role in NSCLC remains unknown. Our study found that DANCR was highly expressed in NSCLC tissues and cell lines. The down‐regulation of DANCR attenuated cancer cell proliferation, migration and invasion and facilitated cancer cell apoptosis. We also discovered that DANCR potentiated mTOR expression *via* competing for miR‐496, which can bind to the 3′‐UTR of mTOR mRNA and negatively regulate it. These findings suggest that DANCR might be a potential oncogene and that its up‐regulation contributes to the progression of ADC.

Recently, the potential role of DANCR in cancer has been reported in several studies; however, these studies showed that DANCR plays opposite roles depending on the tumour type. In colorectal cancer, hepatocellular carcinoma and prostate cancer, DANCR functions as an oncogene; on the contrary, another report indicated that DANCR functions as a tumour suppressor in breast cancer *via* degrading the epigenetic tumour regulator EZH2 13, 14, 15, 16. This phenomenon may explain the variable expression and biological patterns of DANCR in different cancer types. Here, we measured the expression levels of DANCR and found that they were significantly increased in ADC tissues and cell lines. We also revealed that the knockdown of DANCR suppressed tumour cell proliferation, migration and invasion both *in vivo* and *in vitro* and promoted cell apoptosis. These experiments demonstrate that DANCR positively regulates the development of lung cancer.

LncRNAs have diverse roles in different biological processes. For example, a recent study has shown that MALAT‐1 associates with the EZH2 protein to promote osteosarcoma metastasis, whereas another study has reported that MALT‐1 modulates osteosarcoma cells proliferation and migration by directly suppressing miRNA 21, 22. The primary regulatory mechanisms of lncRNA are protein stabilization and miRNA sponging; protein stabilization takes place in both the nucleus and the cytosol while the miRNA sponging takes place only in the cytoplasm 23. Thus, we speculate that DANCR may act through an undiscovered mechanism in lung ADC as it was reported to be more abundant in the cell cytoplasm than in the nucleus 24.

According to the ceRNA hypothesis, lncRNAs could competitively bind to miRNA response elements (MREs) to regulate gene expression, which would be a new post‐transcriptional regulatory mechanism 20, 25, 26, 27. In our study, we confirmed that DANCR functioned as a ceRNA and showed for the first time that miR‐496 was a direct target of DANCR. We found that lung cancer cell motility was obviously inhibited by the down‐regulation of DANCR, and cotransfection with the miR‐496 inhibitor rescued this effect. The luciferase assay indicated that miR‐496 bound to DANCR directly; moreover, the RIP and RNA pull‐down assays further verified that miR‐496 modulated DANCR in a RISC‐dependent manner. These results present evidence that DANCR acts as an endogenous sponge of miR‐496 and that DANCR and miR‐496 negatively regulate each other in ADC. DANCR has been reported to function *via* suppressing protein expression or interacting directly with mRNA, but our results provide evidence of a novel mechanism of action for DANCR 12, 24, 28.

The mTOR signalling pathway is frequently activated in various types of human cancer, and this activation leads to tumour progression 29. High mTOR protein expression levels have been detected in non‐small cell lung cancer (NSCLC) tissues and cell lines, and suppressing mTOR could attenuate the development of cancer 30. Deactivating the mTOR pathway also inhibits cancer metastasis and invasion 31. A recent study proposed that miR‐496 negatively regulated mTOR by targeting two sites within its 3′‐UTR 19. Thus, we suggested that DANCR may affect mTOR expression by competing with miR‐496. Our present study demonstrated that both miR‐496 overexpression and DANCR down‐regulation could reduce mTOR expression, but cotransfection with the miR‐496 inhibitor reversed this decrease in mTOR. This evidence confirms that DANCR may play a tumour suppressor role by sponging miR‐496, which in turn affects its target, mTOR.

Interestingly, we found that DANCR and miR‐496 could suppress each other reciprocally both *in vitro* and *in vivo*, indicating a significant inverse correlation. The phenomenon that miRNA sponging is correlated with a reduction in the inhibited miRNA has been widely reported by previous studies; however, its mechanism remains unclear 27. In addition to the ceRNA function, lncRNAs have also been implicated in post‐transcriptional regulation; for example, lncRNAs can modulate chromatin by regulating its structure 32. LncRNAs can also combine and interact with proteins *via* protein scaffolding 33. It has also been reported that lncRNAs participate in the methylation regulatory network by epigenetically silencing their target gene 34. Due to the complex function of lncRNAs, we speculate that DANCR may down‐regulate miR‐496 *via* an undiscovered regulatory pathway. This hypothesis needs further investigation in the future.

Taken together, our study suggests that DANCR plays an important role in modulating lung ADC progression both *in vitro* and *in vivo* by acting as a ceRNA for miR‐496 to regulate the expression levels of mTOR. DANCR could be used as a diagnostic marker and have therapeutic potential, but a study utilizing a large cohort of NSCLC patients is needed to confirm these suggestions.

## Author contributions

T.R. designed and supervised the research study. Q.C.L. and W.X. performed the experiments. Z.L.G. and S.S. contributed to the data analysis. Z.H.R. enrolled patients and measured the RNA levels in the clinical samples. The manuscript was written by Q.C.L. All authors have seen and approved the final version of the manuscript.

## Conflict of interest

The authors confirm that there are no conflicts of interest.
